# Estimation of 10‐year cardiovascular risk among adult population in western Nepal using nonlaboratory‐based WHO/ISH chart, 2023: A cross‐sectional study

**DOI:** 10.1002/hsr2.1614

**Published:** 2023-10-08

**Authors:** Deekshanta Sitaula, Aarati Dhakal, Sujit K. Mandal, Nisha Bhattarai, Amisha Silwal, Pradeep Adhikari, Shikha R. Gupta, Dhan Khatri, Nimesh Lageju, Bhimsagar Guragain

**Affiliations:** ^1^ Rasuwa Hospital Dhunche Bagmati Province Nepal; ^2^ Department of Community Programs, Dhulikhel Hospital Kathmandu University Dhulikhel Nepal; ^3^ Badegaun Primary Health Center Lalitpur Bagmati Province Nepal; ^4^ Shishuwa Hospital Lekhnath Gandaki Province Nepal; ^5^ Department of Medical Oncology Nepal Cancer Hospital and Research Center Lalitpur Nepal; ^6^ Tulsipur Bhimsen Metro Hospital Tulsipur Dang Nepal; ^7^ Navchetana Kendra Health Care Shastrinagar New Delhi India; ^8^ Bakulahar Ratnanagar Hospital Tandi Nepal; ^9^ Health Office Dhunche Bagmati Province Nepal

**Keywords:** cardiovascular diseases, CVD risk, hypertension, WHO

## Abstract

**Background and Aims:**

Noncommunicable diseases have emerged as a major cause of morbidity and mortality worldwide among which the majority of the deaths are caused by cardiovascular diseases. Estimating the risk of cardiovascular diseases helps eliminate the risk factors and prevent developing cardiovascular diseases in the future. The World Health Organization in association with the International Society of Hypertension has developed risk charts for the estimation of 10‐year risk for cardiovascular diseases. This study aimed to estimate 10‐year cardiovascular risk in the Nepalese population using nonlaboratory‐based charts.

**Methods:**

A hospital‐based cross‐sectional study was conducted among 314 adults aged 40–74 years visiting the outpatient departments of Shishuwa Hospital in western Nepal. Systematic random sampling was used to select the participants. Questionnaire‐guided short interviews, physical examination, and anthropometric measurements were done. The *χ* ^2^ test was used to test the significance and a *p* < 0.05 was considered statistically significant.

**Results:**

As per the risk estimation charts, high cardiovascular risk (20%–30%) was seen in 6.1% of total participants and moderate cardiovascular risk (10%–20%) was found in 29% of participants. The moderate–high risk was significantly higher among male participants compared to females (*p* < 0.01). Of all the participants, 22.0% were current smokers, 17.2% were alcohol users, 61.1% were hypertensive, and 35.7% were diabetics. Smoking tobacco, alcohol use, and hypertension were significantly more prevalent among the male participants. (*p* < 0.01) Adults in the 50–59 years age group had a significantly high prevalence of hypertension (*p* < 0.01), diabetes (*p* = 0.02), and alcohol abuse (*p* = 0.01).

**Conclusion:**

This study shows high cardiovascular risk among adult population in western Nepal. The 10‐year cardiovascular risk score and risk factors were significantly higher among males than females. There seems to be a prompt necessity of health promotion interventions to reduce cardiovascular risk factors and prevent the burden of cardiovascular diseases in Nepal.

## 1 INTRODUCTION

Noncommunicable diseases (NCDs) have become a serious public health burden and leading cause of mortality worldwide. According to World Health Organization (WHO) NCD country profiles, 57 million deaths occurred worldwide in 2016, and 71% of those deaths were caused by NCDs.^1^ According to the latest WHO report, out of 41 million annual deaths due to NCDs, the majority of the deaths, or 17.9 million deaths per year are caused by cardiovascular diseases (CVDs), followed by malignancies (9.3 million), chronic respiratory diseases (4.1 million), and diabetes (2 million along with kidney disease deaths caused by diabetes).^2^ The burden of CVDs is significantly greater in developing and low‐ and middle‐income countries (LMICs) than in high‐income countries.^3^ WHO has reported that more than three‐quarters of CVD mortality takes place in LMICs.^4^ According to Nepal Burden of Disease 2019, NCDs are the primary cause of death, accounting for 66% of all fatalities. Nearly 1 in 6 deaths in Nepal is caused by ischemic heart disease (16%), while chronic obstructive pulmonary disease accounts for 1 in 10 deaths (10% of all deaths).^5^

Multiple risk factors such as smoking, alcohol, unhealthy food habits, physical inactivity, high blood pressure, increased blood cholesterol, and high body mass index (BMI) all combine to cause CVDs. Individual characteristics like age, sex, ethnicity, and history of CVD in the family increase the total risk.^6^ Targeting the risk factors for CVDs can greatly reduce the burden of CVDs and the number of deaths from these conditions.^7^ Risk prediction charts are tools that help to estimate CVD risk by taking into account a variety of risk factors and to classify individuals into high‐ and low‐risk groups.^8^ Multiple CVD risk prediction charts are developed and in use among which Framingham Risk Score (FRS) was the first and was based on Framingham Risk study. Other examples of CVD risk prediction charts are PROCAM, Systematic Coronary Risk Evaluation (SCORE), Reynold Risk Score, QRISK, and so forth. However, the majority of these charts are based on developed high‐income countries thus they may not be applicable in settings with low resources.^9^, ^10^ In 2007, the WHO in collaboration with the International Society of Hypertension (ISH) developed a risk prediction chart for estimating the 10‐year risk of major fatal or nonfatal cardiovascular events. It was developed for 14 WHO epidemiological subregions. In 2019, this chart was updated by the WHO and was presented in two forms; laboratory‐based and nonlaboratory‐based charts. The laboratory‐based risk chart consists of information on age, sex, systolic blood pressure, smoking history, history of diabetes, and total serum cholesterol value while BMI is included in nonlaboratory‐based charts. Information about the history of diabetes and serum cholesterol is not required in nonlaboratory‐based charts to estimate the 10‐year risk of CVDs.^11^ In limited resource settings like Nepal where there is limited availability of laboratory facilities, a nonlaboratory‐based CVD risk prediction chart can be used as an appropriate tool for estimating the risk of CVDs. Many studies have already reported that nonlaboratory‐based WHO/ISH risk prediction charts work almost as accurately as laboratory‐based prediction charts.^9^, ^12^, ^13^

In 2017, Khanal et al.^14^ conducted a study to estimate 10‐year total cardiovascular risk among the Nepalese population using laboratory‐based WHO/ISH risk prediction chart and reported that the low, moderate, and high CVD risk were 86.4%, 9.3%, and 4.3%, respectively. Another laboratory‐based risk estimation study from Nepal reported >10% CVD risk in 14.6% of respondents.^15^ Most of the previous studies in Nepal have used laboratory‐based CVD risk prediction charts to estimate the CVD risk. However, in the present study, we planned to use nonlaboratory‐based risk estimation charts to determine the CVD risk score of adults. As Nepal is a developing and resource‐constrained country, laboratory facilities are not accessible and available to every part of the population. Nonlaboratory‐based risk prediction charts are effective tools to predict the CVD risk of such Nepalese population as they are simple, cost‐effective, noninvasive, and almost identical to laboratory‐based tools in terms of accuracy.^12^, ^13^ The aim of this study was to estimate the total 10‐year CVD risk among the adult population (>40 years) visiting outpatient department of a government hospital in the western region of Nepal using nonlaboratory based WHO/ISH risk prediction tools.

## 2 MATERIALS AND METHODS

### 2.1 Study design and population

A hospital‐based cross‐sectional study was designed to predict 10‐year cardiovascular risk using WHO/ISH chart among the adult population (40–74 years) visiting the outpatient department of Shishuwa Hospital in Pokhara Metropolitan City of western Nepal. Data collection was conducted from March 1 to 30, 2023. Adults aged between 40 and 74 years visiting the outpatient department of the hospital were included in the study. Those with a previous history of myocardial infarction and stroke were excluded from the study.

### 2.2 Study sample

The study site was selected based on convenience sampling. The sample size was calculated based on the prevalence of moderate–high CVD risk among adults aged 40 years and above, as reported by a previous Nepalese study.^15^ With 4% absolute precision, a 95% confidence level, the sample size was calculated to be 300 by using openepi.com (Open‐Source Epidemiologic Statistics for Public Health). Assuming 5% nonresponse rate, the final sample size was 314. Systematic random sampling was used for the selection of the participants. For systematic random sampling, every seventh adult (40–74 years) visiting the outpatient department of Shishuwa Hospital was selected and approached for the study. We informed all the selected individuals about the objectives of our study and took consent from them for the study.

### 2.3 Data collection

After taking informed consent, our team of trained health workers took a short interview using a preverified questionnaire which included questions on their demographics, behavioral risk factors, history of hypertension, diabetes, and CVDs. Then, the blood pressure of the participants was measured, and anthropometric measurements were done.

The blood pressure of the participants was measured at least two times in a sitting position with the help of a digital sphygmomanometer. Before the first measurement, the participants were asked to rest at least for 15 min. The second reading was taken a few minutes after the first measurement. An average of the two readings was taken into analysis.

A portable stadiometer was used to measure the height of the participants and was measured without shoes. The weight of the participant was measured using a digital weighing machine, with minimal clothing and without shoes. BMI was calculated from measured height and weight as weight (kg) per square height (meter).

### 2.4 Study variables

CVD risk score calculated using risk estimation charts was the major outcome variable as well as the dependent variable of the study. Sociodemographic variables (age, gender, education level), blood pressure, smoking history, alcohol abuse, history of diabetes, and BMI were independent variables. BMI was classified according to Asia–Pacific criteria: overweight was defined as BMI between 23 and 24.9 kg/m^2^ and obesity was defined as BMI above 25 kg/m^2^.^16^ Hypertension was defined as having systolic blood pressure ≥140 mmHg and/or diastolic blood pressure ≥90 mmHg or a history of taking antihypertensive medications.

### 2.5 Calculating 10‐year CVD risk using WHO/ISH risk charts

**Figure 1:**
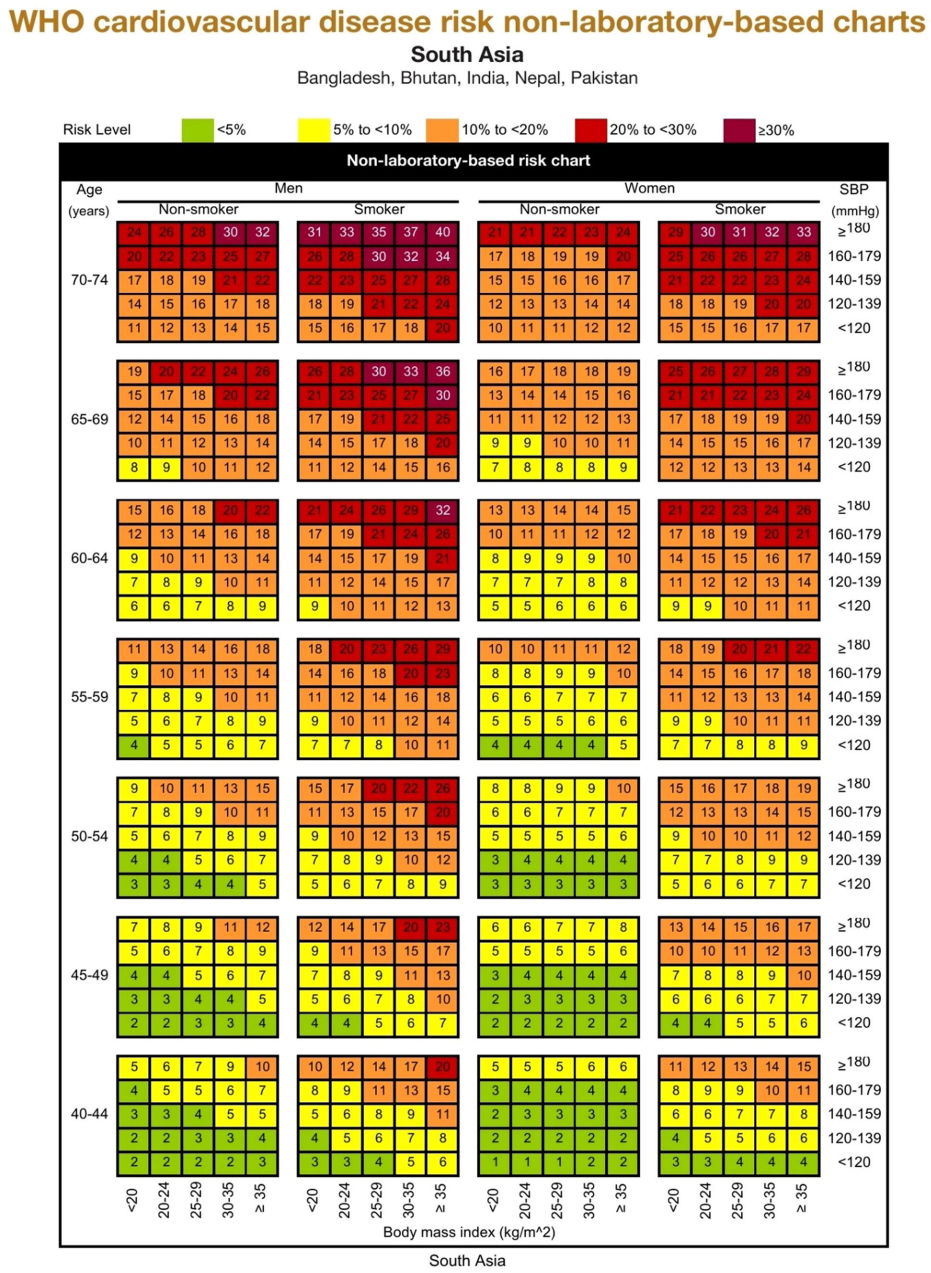
WHO/ISH nonlaboratory‐based CVD risk estimation chart. CVD, cardiovascular disease; ISH, International Society of Hypertension; WHO, World Health Organization.

Ten‐year CVD risk of the participants was calculated using nonlaboratory WHO/ISH CVD risk estimation chart for South East Asian Region (SEAR). The nonlaboratory‐based risk estimation was based on age (40–74 years), gender (male/female), smoking status (smoker/nonsmoker), systolic blood pressure, and BMI (Figure 1). These charts estimate the 10‐year risk of a fatal or nonfatal CVD event (myocardial infarction or stroke) being classified as <10% “low risk,” 10%–19% “moderate risk,” 20%–29% “high risk,” and 30% or more as “very high risk.”^17^

### 2.6 Data analysis

The obtained data were entered in Microsoft Excel. Data analysis was done using SPSS version 22.0. Descriptive analysis was used to determine the frequency and percentage of both dependent and independent variables which were presented in tables and figures. Age and gender‐wise distribution of CVD risk scores were computed. Statistical significance of age and gender with other categorical variables was assessed using the *χ* ^2^ test. *p* Value less than 0.05 was considered to be statistically significant.

## 3 RESULTS

Out of 314 adult participants included in the study, 160 (51.0%) were male and 154 (49.0%) were female. The mean age was 56.6 ± 10.1 years and 31.5% of the total participants were illiterate.

Table 1 shows the distribution of sociodemographic characteristics and CVD risk factors by gender. There was a statistically significant difference in education levels, smoking tobacco, alcohol use, and prevalence of hypertension between male and female participants (*p* < 0.01).

**Table 1:** Distribution of demographic characteristics and CVD risk factors by gender (*n* = 314).

Gender	Male (*n* = 160)	Female (*n* = 154)	Total	*p* Value
	*n* (%)	*n* (%)	*n* (%)	
Age (years)				
40–49	40 (25.0)	54 (35.0)	94 (29.9)	0.16
50–59	42 (26.2)	41 (26.6)	83 (26.4)	
60–69	51 (31.9)	42 (27.3)	93 (29.6)	
≥70	27 (16.9)	17 (11.0%)	44 (14.0)	
Education				
Illiterate	16 (10.0)	83 (55.8)	99 (31.5)	<0.01
Primary level	36 (22.5)	24 (15.6)	60 (19.1)	
Secondary level	91 (56.9)	45 (29.2)	136 (43.4)	
Bachelors and above	17 (10.6)	2 (1.2)	19 (6.0)	
Smoking tobacco	56 (35.0)	13 (8.4)	69 (22.0)	<0.01
Alcohol use	49 (30.6)	5 (3.2)	54 (17.2)	<0.01
Overweight	30 (18.7)	27 (17.53)	57 (18.2)	0.64
Obese	89 (55.6)	89 (57.8)	178 (56.7)	0.64
Hypertension	113 (70.6)	79 (51.3)	192 (61.1)	<0.01
Diabetes	62 (38.7)	50 (32.5)	112 (35.7)	0.25

Abbreviation: CVD, cardiovascular disease.

Table 2 shows the distribution of participants' characteristics by age group. Among the age groups, there was a statistically significant difference in alcohol use (*p*‐value 0.01), the prevalence of hypertension (*p* < 0.01), and the prevalence of diabetes (*p*‐value 0.02).

**Table 2:** Distribution of CVD risk factors by age groups (*n* = 314).

Age group (years)	40–49 (*n* = 94)	50–59 (*n* = 83)	60–69 (*n* = 93)	70 and above (*n* = 44)	Total (*n* = 314)	*p* Value
	*n* (%)	*n* (%)	*n* (%)	*n* (%)	*n* (%)	
Gender						
Male	40 (42.5)	42 (50.6)	51 (54.8)	27 (61.4)	160 (51.0)	0.16
Female	54 (57.5)	41 (49.4)	42 (45.2)	17 (38.6)	154 (49.0)	
Smoking tobacco	16 (17.0)	23 (27.7)	16 (17.20)	13 (29.50)	68 (21.7)	0.12
Alcohol use	9 (9.6)	23 (27.7)	13 (13.9)	9 (20.4)	54 (17.2)	0.01
Overweight	12 (12.7)	14 (16.8)	21 (22.5)	10 (22.7)	57 (18.2)	0.61
Obese	61 (64.9)	50 (60.2)	46 (49.4)	21 (47.7)	178 (56.7)	0.61
Hypertension	43 (45.7)	54 (65.1)	59 (63.4)	36 (81.8)	192 (61.1)	<0.01
Diabetes	23 (24.4)	37 (44.6)	39 (41.9)	13 (29.5)	112 (35.7)	0.02

Abbreviation: CVD, cardiovascular disease.

**Figure 2:**
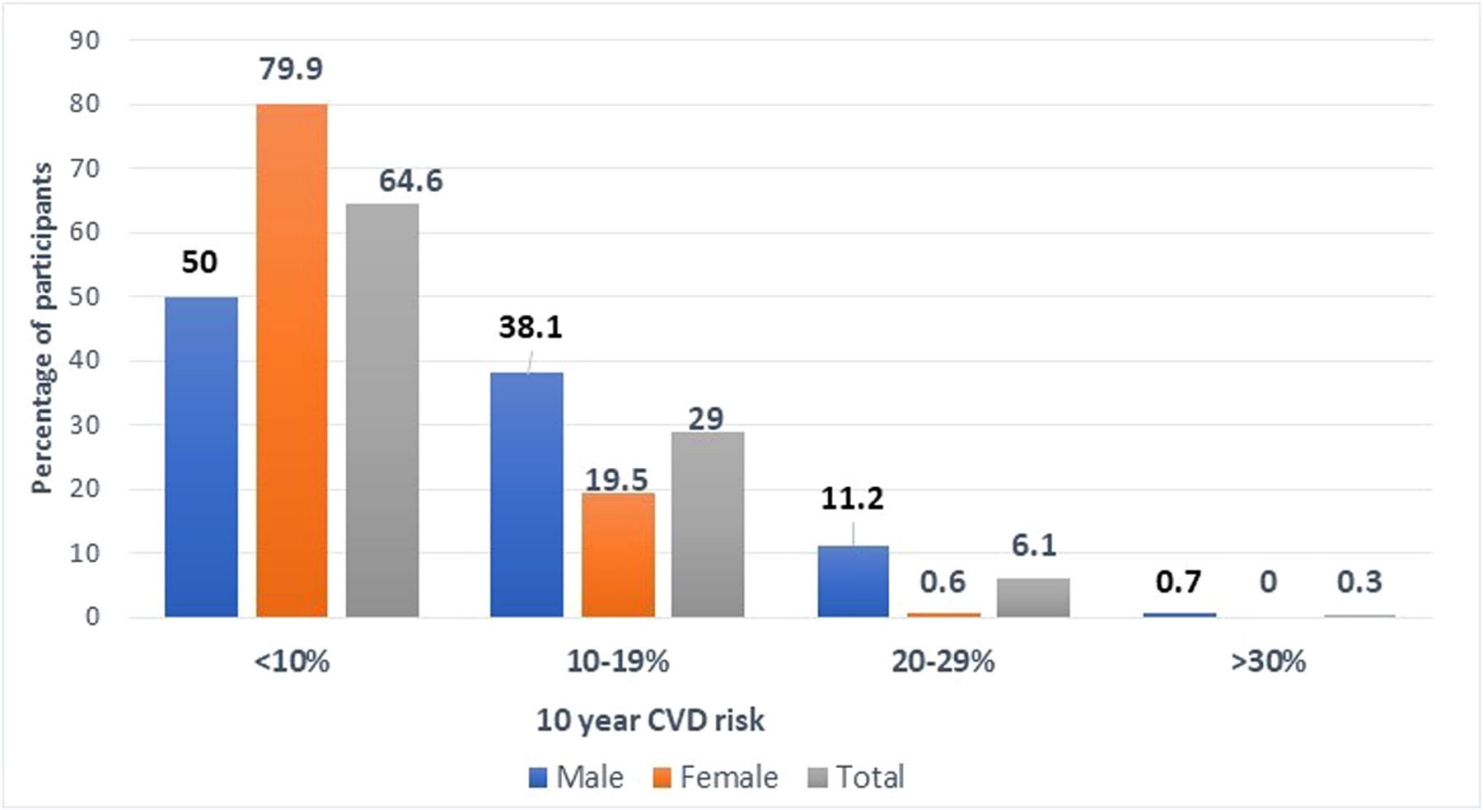
10‐year cardiovascular risk levels distribution by gender (*n* = 314) (*p* < 0.01). CVD, cardiovascular disease.

Figure 2 shows the gender‐wise distribution of 10‐year CVD risk. Moderate–high CVD risk is significantly higher (*p* < 0.01) among males compared to females. Cardiovascular risk according to the age group is shown in Table 3. Very high and high cardiovascular risk (>20%) was seen mostly among people aged 70 years and above. 49.4% of participants in the 60–69 years age group, and 63.6% of participants above 70 years had moderate cardiovascular risk (10%–20%).

**Table 3:** Distribution of CVD risk scores by age (*n* = 314) (*p* < 0.01).

Age (years)	40–49	50–59	60–69	≥70	Total
CVD risk	*n* (%)	*n* (%)	*n* (%)	*n* (%)	*n* (%)
<5%	76 (80.8)	17 (20.4)	0 (0.0)	0 (0.0)	93 (29.6)
5%–9%	14 (14.9)	53 (63.9)	43 (46.2)	0 (0.0)	110 (35.0)
10%–19%	4 (4.3)	13 (15.7)	46 (49.4)	28 (63.6)	91 (29.0)
20%–29%	0 (0.0)	0 (0.0)	4 (4.4)	15 (34.1)	19 (6.1)
≥30%	0 (0.0)	0 (0.0)	0 (0.0)	1 (2.3)	1 (0.3)

Abbreviation: CVD, cardiovascular disease.

## 4 DISCUSSION

CVDs mainly result due to atherosclerosis, which is preceded by many identifiable factors. These include age, gender, smoking status, systolic blood pressure, and cholesterol levels. People who exhibit these risk factors are at higher risk of developing CVD in the future compared to those who do not have these risk factors.^18^ Individual risk factors however can predict the risk of CVD; however, the combined approach has been more promising.^18^ Although various cardiovascular risk prediction models like FRS, SCORE, and WHO/ISH CVD risk prediction charts have been put forward by various organizations, WHO/ISH risk prediction charts are recommended for the resource‐poor setting like Nepal as it provides charts both with and without cholesterol.^19^

The current study showed that 35.4% of the participants had an elevated 10‐year risk of CVDs (>10%). In contrast to the published native and international studies, our study shows an increased 10‐year risk of CVD in the general population aged above 40 years. Khanal et al. in 2017 reported that 13.6% population had a moderate to high risk of CVDs. This finding was consistent with a similar study by Dhungana et al. in 2015, which showed an elevated risk of 14.6% in the residents of Kathmandu, Nepal.^14^, ^15^ Our study also revealed that 6.1% of the population had 10 years CVD risk of more than 20%. This is also slightly higher than the previously reported studies including Khanal et al.^14^ and Mendis et al.^20^ which showed that 4.3% and 3.9% of the population had high‐risk CVD, respectively. This study also suggests an increased burden of moderate to high (>10%) CVD risk in Nepal when compared to India (17%), China (3.9%), Pakistan (20.8%), Sri Lanka (5%), and Iran (6.2%).^17^, ^20^ We also found that 49.3% of males had moderate to high risk of CVDs, meanwhile risk in females counter only 20.1%. This is in accordance with previous studies reporting an increased risk of CVD in males than in females.^21^

As we found that the estimated risk is higher than the previous studies done in Nepal and internationally, we suspect an increasing trend in CVD risk in the Nepalese population. Also, the present study is a hospital‐based study conducted among the adult population visiting the outpatient department of a suburban hospital, which can be the reason behind the significantly more prevalence of high‐CVD risk compared to previous community‐based studies.^14^, ^15^ Meanwhile, the possibility of the WHO/ISH chart overestimating the risk in the Nepalese population should also be taken into consideration. The findings of a study done in 2014 have also reflected that the WHO/ISH charts were unable to stratify the risk in the Malaysian population compared to FRS and SCORE prediction model. The findings of the study revealed that many of the participants were falsely categorized into low‐risk groups.^19^ However, in another study, the “without cholesterol” version of the WHO/ISH chart showed good agreement with the FRS in predicting CVD risk in comparison to the “with cholesterol” version and recommended the “without cholesterol” version as a good option in predicting CVD risk in low resource settings.^22^ These findings further point out the importance of further validation of WHO/ISH risk estimation charts to make it a gold standard for resource‐poor settings.

In the present study, 61.1% of the participants were hypertensive and 35.7% were diabetic. This finding is remarkably higher than previously reported Nepalese studies on cardiovascular risk factors.^14^, ^15^, ^23^ Similarly, the prevalence of hypertension and diabetes as reported by this study is higher in comparison to previous studies reported from India,^24^ Bangladesh,^25^ and Vietnam.^17^ Likewise, the prevalence of obesity is also higher in the present study than in these studies.^14^, ^17^, ^23^, ^24^, ^25^ This may indicate increased risk factors for CVDs among the adult population in Nepal. Also, our study is conducted in a hospital‐based setting which might have increased the prevalence of high CVD risk and other risk factors like diabetes, hypertension, and obesity.

As per the WHO guidelines, there is no clear cut‐off for initiating the intervention for CVD risk but it suggests intensive intervention for risk >30% and >40% in medium and low resource settings, respectively. Our study suggests that if a threshold of 30% is to be taken, only a handful of the population will benefit from intervention. However, if we reduce the intervention threshold to 20%, a substantial proportion of the population can be prevented from cardiovascular morbidity.^18^ This study also throws some light on the proportion of the population who are in need of lifestyle and appropriate pharmacological intervention.

## 5 LIMITATIONS

There are few limitations to this study. This study was conducted in a hospital setting rather than from the general population, which may result in Berkons' bias. Also, this limits the generalizability of the study and may not reflect the true community prevalence. Although the WHO/ISH chart that we used for estimating CVD risk is an effective tool for low resource settings, it has its own limitations. It lacks adjustment for the people who are taking antihypertensive medications during the calculation of CVD risk. Additionally, this tool underestimates CVD risk among those with target organ damage, and diabetic patients with complications.^20^

## 6 CONCLUSIONS

The present study found that there was high prevalence of moderate–high CVD risk among the adult population in western Nepal, especially in males. CVD risk factors like smoking tobacco, harmful use of alcohol, and hypertension were found significantly more in males than females. The findings of the study highlight an immediate need of health promotion interventions to reduce the risk factors and prevent the burden of CVDs in Nepal. Further comprehensive studies with larger study populations and more efficient risk prediction tools for determining CVD risk are recommended.

## AUTHOR CONTRIBUTIONS

**Deekshanta Sitaula**: Conceptualization; formal analysis; funding acquisition; methodology; supervision; writing—original draft; writing—review & editing. **Aarati Dhakal**: Conceptualization; data curation; formal analysis; investigation; methodology; project administration; resources; writing—original draft. **Sujit K. Mandal**: Data curation; methodology; project administration; validation; visualization; writing—original draft. **Nisha Bhattarai**: Data curation; software; validation; visualization; writing—original draft. **Amisha Silwal**: Data curation; funding acquisition; supervision; validation; writing—original draft; writing—review & editing. **Pradeep Adhikari**: Data curation; writing—original draft. **Shikha R. Gupta**: Data curation; writing—original draft; writing—review & editing. **Dhan Khatri**: Data curation; writing—original draft; writing—review & editing. **Nimesh Lageju**: Data curation; writing—original draft. **Bhimsagar Guragain**: Supervision; writing—original draft; writing—review & editing.

## CONFLICT OF INTEREST STATEMENT

The authors declare no conflict of interest.

## ETHICS STATEMENT

Ethical approval for the study was taken from Nepal Health Research Council (NHRC) (Ref no. 2503). Before data collection, all the participants were informed about the study objectives and informed consent was taken from them.

## TRANSPARENCY STATEMENT

The lead author Deekshanta Sitaula affirms that this manuscript is an honest, accurate, and transparent account of the study being reported; that no important aspects of the study have been omitted; and that any discrepancies from the study as planned (and, if relevant, registered) have been explained.

## Data Availability

The raw data will be provided on reasonable request through an email to the corresponding author.
